# PEGylated Magnetite/Hydroxyapatite: A Green Nanocomposite for T2-Weighted MRI and Curcumin Carrying

**DOI:** 10.1155/2022/1337588

**Published:** 2022-05-27

**Authors:** Nahideh Gharehaghaji, Baharak Divband

**Affiliations:** ^1^Department of Radiology, Faculty of Allied Medical Sciences, Tabriz University of Medical Sciences, Tabriz, Iran; ^2^Dental and Periodontal Research Center, Tabriz University of Medical Sciences, Tabriz, Iran

## Abstract

**Background:**

The design of new magnetic resonance imaging (MRI) contrast media with chemotherapy drug-carrying capacity has an important role in diagnostic and therapeutic purposes. This study aimed to synthesize a polyethylene glycol (PEG)-coated magnetite/hydroxyapatite nanocomposite as an MRI contrast agent investigate its curcumin loading/release properties and consider the cytotoxicity effect of the curcumin-loaded nanocomposite on different cell lines.

**Materials and Methods:**

PEGylated magnetite/hydroxyapatite (PMHA) nanocomposite was synthesized and characterized using *X*-ray diffraction, Fourier transform infrared spectroscopy, transmission electron microscopy, vibrating sample magnetometry, and energy dispersive *X*-ray analysis. MTT assay was performed to consider the A549, MCF-7, and MRC-5 cells toxicity of the PMHA and the curcumin-loaded nanocomposite. The r2 relaxivity of the nanocomposite was determined by an MRI device. The curcumin loading and its release from the nanocomposite at pH of 7.4 and 5.5 were investigated.

**Results:**

The spherical nanocomposite showed an average size of 20 nm and a superparamagnetic property. PMHA nanocomposite was highly cytocompatible, while the curcumin-loaded nanocomposite showed significant cytotoxicity for A549 and a much higher toxic effect on MCF-7 cancer cells. The r2 relaxivity was measured as 120 mM^−1^S^−1^. The curcumin loading capacity of PMHA was 1.9 mg/g, and the curcumin release profile showed a pH-dependent sustained release of the anti-cancer drug that was higher for pH of 5.5.

**Conclusion:**

The high r2 relaxivity of PMHA nanocomposite and sustained release of curcumin from the loaded one at the pH of tumor environment suggest that the nanocomposite is a potential candidate for T2-weighted MRI and cancer treatment.

## 1. Introduction

Magnetite (Fe_3_O_4_) nanoparticles have been used as magnetic resonance imaging (MRI) contrast agents due to their superparamagnetic behavior, surface chemical property [1], small size, stability, and low toxicity [2]. Compared to gadolinium-based contrast agents of MRI, which can lead to nephrogenic systemic fibrosis and brain deposition [3], magnetite nanoparticles provide more biocompatibility because of the storage of their degraded products by ferritin [4]. During MR imaging, the nanoparticles are exposed to a strong external magnetic field and create local magnetic field inhomogeneities, decreasing signal intensity due to rapid spin–spin interactions and T2 shortening. Therefore, they have been used as negative contrast agents in MRI [5, 6]. The magnetic resonance behavior of magnetite nanoparticles is mainly influenced by their hydrodynamic size and coating material type, and thickness [7]. Magnetite nanoparticles have been coated by different biocompatible materials for various biomedical applications. Among the different coating types, hydroxyapatite (HA) and polyethylene glycol (PEG) have unique places as inorganic and organic coatings, respectively.

HA Ca_10_(PO_4_)_6_(OH)_2_ is a calcium phosphate bioceramic with a Ca/P ratio similar to natural bone and teeth [8]. Due to its biocompatibility and stability, HA has attracted great interest in different biomedical applications [9], such as bone tissue engineering [10], antibacterial activity [11], magnetic hyperthermia [12], MRI contrast agent development [13], gene delivery [14], chemotherapeutic drug delivery [15], and cancer theranostics [16]. PEG is a hydrophilic biocompatible polymer commonly utilized in biomedical applications [17–22]. It is used as coating material around magnetite nanoparticles in MRI and drug delivery applications [23]. Coating nanostructures with PEG plays a crucial role in improving their chemical and biophysical properties [17]. In the PEGylation process, PEG is conjugated to a therapeutic drug, leading to the improved water solubility of hydrophobic chemotherapeutic drugs, increased blood half-life of the nanostructures, and provided the ability of specific tumor targeting [24].

Among the various chemotherapeutic drugs, curcumin is a hydrophobic drug that shows anti-cancer effects by preventing carcinogenesis, tumor growth, and angiogenesis [25]. It also has other pharmaceutical advantages, such as anti-inflammatory and antiviral activity. However, poor water solubility and bioavailability of curcumin limit its clinical application. Loading curcumin into a nanocarrier such as magnetic nanoparticles can overcome these limitations and improve therapeutic efficiency [26].

So far different studies have been carried out using two or more of the nanomagnetite, HA, PEG, and curcumin for MRI or drug delivery applications. Some instances are as follows: The Fe_3_O_4_ core and PEG shell nanoparticles that conjugated with doxorubicin (SPIO-PEG-D) were developed as a potential drug delivery system for MRI monitoring of the tumor and chemotherapy [27]. In another study, the rod-like shape magnetic mesoporous HA nanocomposite coated with PEG and folic acid were used as a carrier for doxorubicin delivery. The nanocomposite was introduced as a multifunctional magneto-responsive platform for cancer treatment [28]. In one study, the Fe_3_O_4_ nanoparticles decorated with PEGylated curcumin (MNP@PEG-Cur) were introduced for dual-targeted drug delivery [29]. Results of other studies showed the potential of magnetized hydroxyapatite nanocrystallites [30], Fe-modified calcium deficient hydroxyapatite nanoparticles [31], and Fe_3_O_4_@HAp core-shell nanoparticles [13] to be used as MRI contrast agents. However, they did not report the r2 relaxivity values. In another study, the r2 values of superparamagnetic mesoporous silica/hydroxyapatite hybrid nanocomposite (MSFeHA) was calculated, but they did not investigate anticancer drug loading and release [32]. Both MRI relaxivity and curcumin delivery of rod-shaped magnetic HA nanocomposite were investigated in the Kermanian et al. study [33]. Also, in our previous work, the transverse relaxivity and curcumin loading/release of spherical-shaped porous calcium phosphate-coated iron oxide nanoparticles were studied [34]. However, according to our knowledge, MRI and curcumin loading/release properties of PEG-coated magnetite/hydroxyapatite nanocomposite have not been investigated.

Since PEG coating provides high biocompatibility and decreases the hydrophobicity of anticancer drugs surface [35], we aimed to consider the potential of PEGylated magnetite/hydroxyapatite nanocomposite for T2-weighted MRI and curcumin carrying in the present study. Besides, we investigated the cytotoxicity effect of the curcumin-loaded nanocomposite on A549 human alveolar adenocarcinoma and MCF-7 human breast cancer cell lines as cancer groups, and MRC-5 human normal lung cell line and compared the results with the free curcumin. Therefore, a potential bi-functional nanocomposite can be introduced for diagnostic and therapeutic purposes.

## 2. Materials and Methods

This research work was an experimental and *in vitro* study. All of the materials used in the study were purchased from Sigma Chemical Co.

### 2.1. Synthesis of Magnetic HA (MHA) and PEGylated Magnetic HA (PMHA)

According to our previous study, MHA nanocomposite was synthesized by the coprecipitation method with some modifications [34]. First, under N_2_ atmosphere, 30 ml aqueous solution of FeCl_2_.4H_2_O and FeCl_3_.6H_2_O with a molar ratio of 2 : 1 was prepared, and 25% NH_4_OH solution was added dropwise under a magnetic stirrer (300 rpm) for 30 min at 80°C. The pH of the resulting solution was adjusted at 10 and stopped heating. Then, the aqueous solution of Ca(NO_3_)_2_.4H_2_O and (NH_4_)_2_HPO_4_ was gradually added to the as-prepared suspension, and by adding NH_4_OH solution, the pH of the whole solution reached 11. The precipitation was formed simultaneously and stirred for 30 min at room temperature and heated up to 90°C for 2 h, stayed overnight, washed, dried in vacuum, and calcinated at 400°C.

For the PEGylation process, a suspension of MHA nanoparticles was ultrasounded for 1 h, and a solution of PEG 4000 was slowly added to the suspension under continued ultrasound and heated up to 70°C for 2 h. Then the suspension was stirred overnight under reflux. The particles were washed several times to eliminate unbounded PEGs, then the product dried at room temperature and was named PMHA.

### 2.2. Physicochemical Characterization Tests

Analysis of the crystal structure of PMHA nanocomposite was obtained using a Philips *X*-ray diffractometer. The FTIR spectrum was recorded by a Bomem MB-Series FTIR spectrophotometer (Hartman–Braun, USA) in the range of 400 to 4000 cm^−1^ using KBr pellets. The shape and size of the nanocomposite were determined by the use of a transmission electron microscope (TEM) device (Zeiss LEO 912 Omega). Investigation of the magnetic properties of PMHA was carried out using a vibrating sample magnetometer (VSM model 7400) at room temperature. The applied magnetic field was between −20000 and +20000 Oe.

### 2.3. In Vitro Curcumin Loading and Release

PMHA was considered as a host for the encapsulation of curcumin by a soaking procedure. Briefly, 1 g of the nanocomposite was dispersed in distilled water, mixed with curcumin dissolved in ethanol (2 mg/mL), and stirred for 48 h at room temperature. Then, the solution was centrifuged for 20 min at 8,000 rpm. The products were dried in an oven at 60°C for 12 h and named CUR@PMHA. The extent of curcumin encapsulation in carriers was calculated considering the absorption of the supernatant at *λ* = 418 nm by UV–visible spectrophotometer (Jenway 6305).

The in vitro study of curcumin release from CUR@PMHA was based on the previous study at pH 7.4 and 5.5 [36]. The phosphate-buffered saline (PBS) with 0.5% Tween 80 was prepared. A dialysis bag with a cutoff of 12 kDa was used to place 20 mg of CUR@PMHA and introduce it to 20 mL of PBS with stirring at 37°C. To determine the concentration of curcumin in dialysate and obtain the time-dependent release profile of the anticancer drug, at the time intervals, taking out 1 mL of dialysate and replacing it with 1 mL of the solution of fresh buffer was performed. During the process, the temperature was maintained at 37°C. The measurements were carried out at *λ* = 418 nm by a UV–V is spectroscope (Jenway 6305).

### 2.4. Cell Culture and In Vitro MTT Assay

Human alveolar adenocarcinoma (A549) and breast cancer (MCF-7) cell lines as cancer groups and human normal long cell line (MRC-5) were obtained from the cell bank of Pasteur Institute (Karaj). The cells were cultured in RPMI-1640 medium containing 10% fetal bovine serum (FBS) and 1% penicillin and streptomycin antibiotics, and then the cells were incubated with 5% CO_2_ at 37°C [37].

The cell toxicity of MHA, PMHA, CUR@MHA, CUR@PMHA, and free curcumin was investigated on A549, MCF-7 cancer cells, and MRC-5 normal cells. The cells (2 × 10^4^ cells/well) were incubated in the 96-well plates with 200 *µ*L/well of the supplemented cell culture medium for 24 h at 37°C and 5% CO_2_. The cells were treated with different concentrations of the samples. After incubation, the media were eliminated, and the wells were washed using PBS with a pH of 7.4. To measure the cellular proliferation, 50 *μ*L of MTT solution (2 mg/mL), and 150 *μ*L culture medium were added to each well. Then, the A549 cells were incubated for 24 h at 37°C and 5% CO_2_. As the next step, the media was removed from the wells, and dimethyl sulfoxide (200 *µ*L) and Sorenson solubilizer buffer (25 *µ*L) were added to each well. The absorbance of the samples was read at 570 nm wavelength using an ELISA plate reader (BioTek, Bad Friedrichshall, Germany).

The IC_50_ values were determined to compare the cytotoxicity of CUR@MHA, CUR@PMHA, and free curcumin in A549, MCF-7, and MRC-5 cell lines.

GraphPad Prism 8.0.2 software was used for statistical analysis of the data. The results were presented as mean ± standard deviation (SD). Statistical significance was analyzed by one-way analysis of variance (ANOVA), and *p* < 0.05 was considered to be statistically significant.

### 2.5. MR Phantom Imaging

MRI of PMHA nanocomposite was performed using a 1.5 T clinical MR imager (Siemens, AG 2009 AVANTO, Germany) on glass tubes containing the nanocomposite suspension with the different Fe^3+^ concentrations. The T2-weighted MR images were taken using a multispin echo sequence with 3000 ms repetition time, ranging from 22 to 352 ms echo times, 5 mm slice thickness, 120 × 90 mm^2^ field of view in the 128 × 128 matrix size. MATLAB software was used for the following steps:

- Measuring signal intensity on Dicom image series by introducing a similar size circular region of interest in the middle of each tube.
- Drawing descending exponential fits of the T2 relaxation times.
- Calculating the inverse of T2 relaxation time (1/T2) values for each sample.

Then the r2 relaxivities were extracted from the slope of the inverted T2 relaxation times of PMHA versus Fe^3+^ concentration graph.

## 3. Results

### 3.1. Characterization Tests

**Figure 1:**
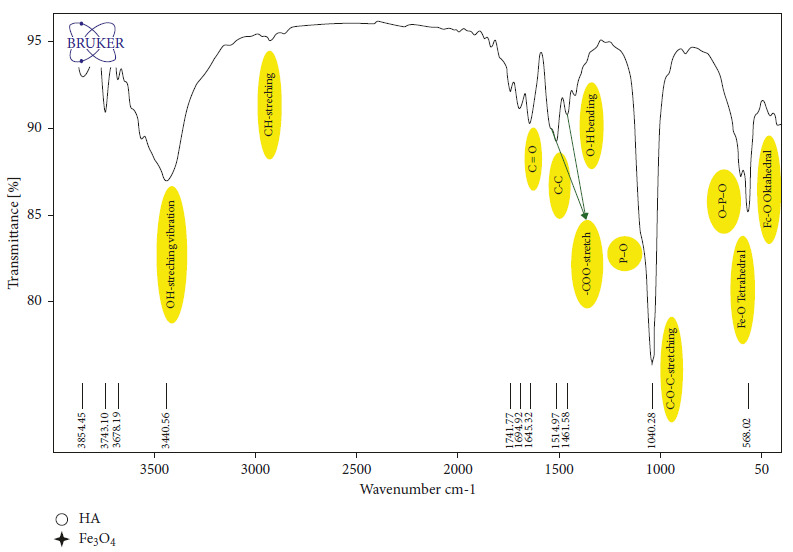
XRD patterns of MHA and CUR@PMHA.

The XRD patterns of MHA and CUR@PMHA are shown in Figure 1. The characteristic peaks of hydroxyapatite and Fe_3_O_4_ were identified [34]. Decreasing the intensity of diffraction peaks of HA and Fe_3_O_4_ was seen following a physical PEGylation process.

**Figure 2:**
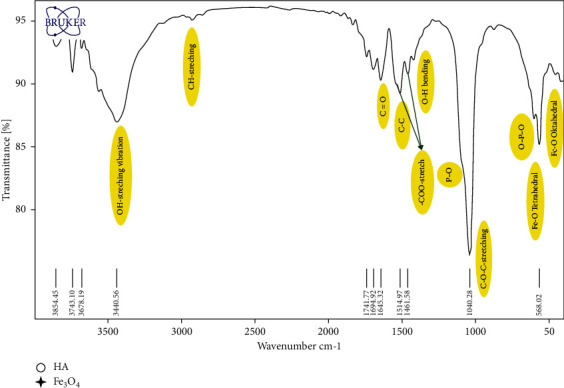
FTIR of CUR@PMHA nanocomposite. The peaks related to the functional groups corresponding to the Fe_3_O_4_, hydroxyapatite, and PEG are observed.

The FTIR spectrum of the functional groups in CUR@PMHA is presented in Figure 2. The main characteristic peaks of Fe_3_O_4_ (the bending Fe-O bond) are observed at wavenumbers of 568 cm^−1^ (in the tetragonal position) and 430 cm^−1^ (in the octahedral position), and the stretching Fe-O bond at 1384 cm^−1^ [38]. The peaks at around 568 and 600 cm^−1^ wavenumber region, corresponding to phosphate vibrations (O–P–O) and at about 1120 cm^−1^ wavenumber region, belonging to phosphate groups (P–O) of hydroxyapatite. The presence of PEG coating on HA was proved by the C-O-C stretch bond (1040 cm^−1^), C-H stretch bond (2900 cm^−1^), and -COO-stretch bond (1570 cm^−1^ and 1461 cm^−1^), the shift appeared in these peaks due to loading of curcumin [38, 39]. The sharp and broad peaks of the OH groups (OH-stretching vibration) of PEG and hydroxyapatite appear around 3440 cm^−1^.

**Figure 3:**
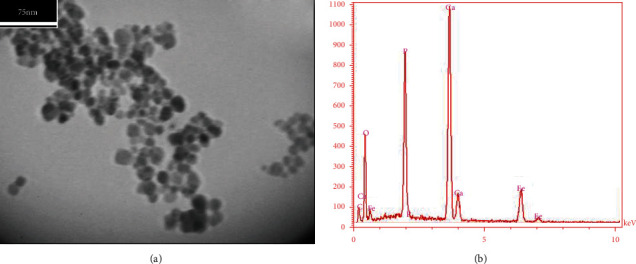
(a) TEM image shows the spherical shape with 20 nm size of PMHA, and (b) EDX graph demonstrates the presence of Ca, OP, Fe, and C in PMHA nanocomposite.

Based on the TEM image (Figure 3(a)), the size of the spherical shape PMHA was measured around 20 nm. The composition of PMHA and the composite elements analyzed using EDX is shown in Figure 3(b). According to the EDX graph, Ca (19.33%), O (52.25%), P (11.48%), Fe (2.85), and C (13.89%) were presented in PMHA.

**Figure 4:**
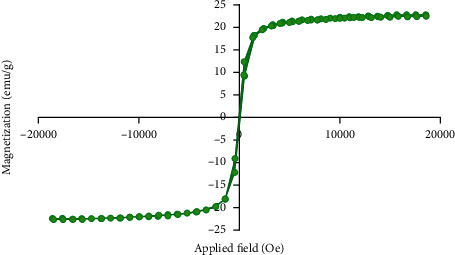
Magnetization versus applied magnetic field curve for PMHA nanocomposite. The shape of the curve implies the superparamagnetic behavior of the nanocomposite.

Figure 4 illustrates the magnetization versus applied field curve for PMHA nanocomposite. No hysteresis loop, remnant magnetization, and coercivity were seen in the curve.

### 3.2. In Vitro Curcumin Loading Capacity and Release Profile

The adsorption capacity of PMHA was 1.9 mg/g (curcumin/nanocomposite), reached a maximum equilibrium after 2 h exposure with curcumin solution.

**Figure 5:**
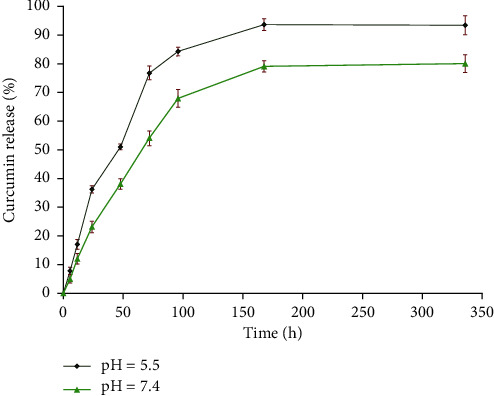
The curcumin release profiles from CUR-PMHA nanocomposite at different pH conditions (pH of 7.4 and 5.5) at 37°C.

Figure 5 shows the in vitro release profiles of curcumin from CUR@PMHA nanocomposite at pH of 7.4 and 5.5. A biphasic release profile including rapid release at first and sustained release following was seen for both pHs, higher for pH of 5.5. The release of curcumin from CUR@PMHA (pH of 5.5) reached 51 and 76% at 48  and 72 h, respectively, while it was 38 and 54% at the same times for pH of 7.4, respectively.

### 3.3. MTT

**Figure 6:**
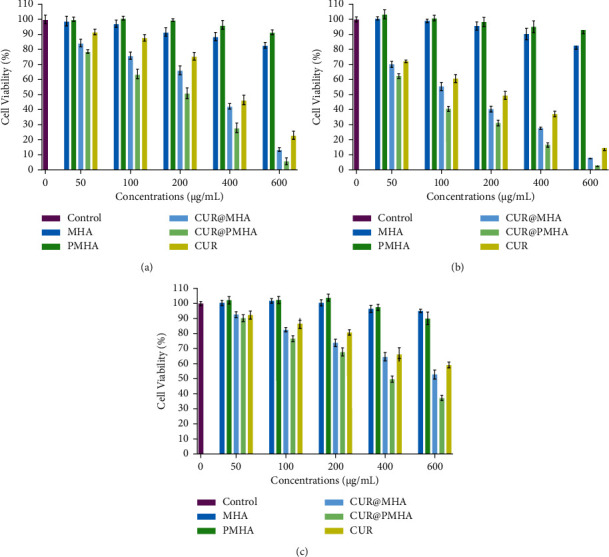
MTT cell viability results for (a) A549 and (b) MCF-7 cancer cells and (c) MRC-5 normal cells treated with different concentrations of MHA, PMHA, CUR@MHA, CUR@PMHA, and free curcumin at 24 h. All data were reported as the mean ± SD (*n* = 4).

The cell toxicity of A549, MCF-7, and MRC-5 cells treated with different concentrations (50, 100, 200, 400, and 600 *µ*g/mL) of MHA, PMHA, CUR@MHA, CUR@PMHA, and free curcumin at 24 h is seen in Figure 6(a)–6(c), respectively. Both MHA and PMHA nanocomposites showed considerable cell viability for the treated cells at all concentrations with higher compatibility for PMHA.

The cell viability of A549, MCF-7, and MRC-5 was reduced in concentration-dependent manner when treated with CUR@MHA, CUR@PMHA, and free curcumin. The IC_50_ values in A549 group were calculated to be 335 ± 2.1, 209 ± 3.5, and 376 ± 3.1 *µ*g/mL for CUR@MHA, CUR@PMHA, and free curcumin, respectively (Figure 6(a)). In MCF-7 cell line, the IC_50_ values were determined to be 136 ± 2.2, 78 ± 1.6, and 194 ± 2.7 *µ*g/mL for CUR@MHA, CUR@PMHA, and free curcumin, respectively (Figure 6(b)) that were lower than A549 cells.

As seen in Figure 6(c), in MRC-5 normal cells, the calculation of the IC_50_ value only was feasible for CUR@PMHA (397 ± 2.5 *µ*g/mL). The highest amount of IC_50_ among the tested groups was obtained for this nanomaterial.

### 3.4. MRI

**Figure 7:**
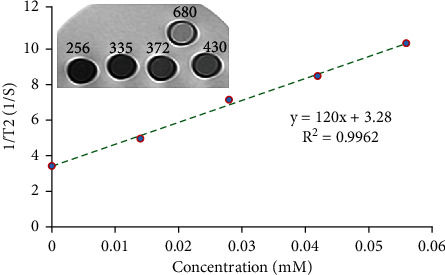
The r2 relaxivity graph of PMHA nanocomposite shows a linear relationship between the inverse of the T2 relaxation times and Fe concentrations, inset: the T2-weighted MR image of the nanocomposite samples with various Fe concentrations. Reduction in the signal intensity with increasing the Fe concentration is seen.

The T2-weighted MR images of PMHA samples demonstrated concentration-dependent signal changes from the high-to-low signal intensity. The inset of Figure 7 shows a T2-weighted MR image prepared with TR/TE of 3000/44 ms. Signal drop from 430 for the lowest concentration of PMHA to 256 for the highest one is seen in the figure. The values were 1.6 and 2.7 times lower than the zero concentration with a signal intensity of 689, respectively. There was seen a linear relationship between 1/T2 and iron concentration. The relaxivity of the nanocomposite was calculated 120 mM^−1^S^−1^.

## 4. Discussion

### 4.1. Characterization Tests

According to Figure 1 which shows the XRD patterns of MHA and CUR@PMHA, following a physical PEGylation process, the intensity of diffraction peaks of HA and Fe_3_O_4_ was decreased due to the effect of both PEG and curcumin that obstructed their crystal plane. Therefore, the XRD results confirmed the existence of an organic coat on the MHA without damaging most of its crystals [39].

As is seen in the FTIR spectrum of CUR@PMHA (Figure 2), the peaks related to the functional groups corresponding to the Fe_3_O_4_, hydroxyapatite, and PEG are observed. The observed bands shows phosphate and hydroxyl vibrational modes existence for HA formation. The experimentally observed bands are in agreement with the reported HA values. The presence of PEG and curcumin on HA does not play any role in the structural deformation of HA.

Based on the TEM results (Figure 3(a)), the spherical shape of PMHA helps to easily transfer the nanocomposite in blood vessels. Since the size of PMHA (around 20 nm) is larger than the glomerular filtration threshold, it is not rapidly excreted through the kidneys [40]. Hence, the nanocomposite can stay longer time in blood circulation, providing more time for MR imaging.

EDX results (Figure 3(b)) showed the presence of Ca, O, P, Fe, and C in PMHA as the composite elements, demonstrating the successful synthesis of the nanocomposite. The Ca/P ratio estimated from the EDX was 1.68, close to the stoichiometric ratio (1.67) of the natural HA [41], which proved the successful synthesis of HA with the proposed process.

According to Figure 4, the absence of hysteresis loop, remnant magnetization, and coercivity in the magnetization versus applied field curve for PMHA nanocomposite confirms its superparamagnetic property. This means the nanocomposite shows only the magnetic property when placed in the magnetic field. The superparamagnetic property also prevents aggregation of the nanocomposite and it is essential for intravenous administration of the iron oxide-based nanostructures [42].

### 4.2. Curcumin Loading Capacity and Release Profile

In this study, a PEG with a molecular weight of 4000 kDa was used to achieve a high encapsulation efficiency for the chemotherapeutic drug. This selection was based on the results of Akbarzadeh et al. study about the doxorubicin-loaded magnetite nanoparticles with PLGA-PEG copolymers which showed the highest anticancer drug efficiency using PEG 4000 compared to the PEG 2000 and 3000 [43].

The in vitro release profile of curcumin from CUR@PMHA nanocomposite at physiologic pH of 7.4 and acidic pH of 5.5 showed a biphasic release profile for both pHs that was higher for pH of 5.5 (Figure 5), indicating the pH-dependent release of the drug. Normal tissues are in physiologic pH while tumoral tissues are in acidic condition. Since pH of 5.5 is similar to the environment of tumor cells, the findings imply that CUR@PMHA can be used for cancer cells treatment. The pH-dependent curcumin release not only is suitable for cancer treatment but also reduces the side effects of the drug in normal tissues.

In our earlier study with the curcumin-loaded calcium phosphate-coated iron oxide nanoparticles, curcumin release was investigated only at pH 7.4 [34], which showed lower curcumin release at this pH than the present study. This is due to the ability of curcumin to chelate with metal ions that provides curcumin adherence to the calcium phosphate matrix (calcium ions). On the other hand, PEG coating around HA provides curcumin hydrophilicity and improves its aqueous solubility, leading to a higher release for CUR@PMHA at similar conditions of time and pH [35].

### 4.3. MTT

MTT assay in this study was performed to consider the cytocompatibility of MHA and PMHA nanocomposites and to investigate the toxicity of CUR@MHA, CUR@PMHA, and free curcumin in A549, MCF-7, and MRC-5 cell lines based on IC_50_ determination.

As seen in Figure 6, PMHA nanocomposite showed high cell viability for all three cell lines at all concentrations. Although MHA was also cytocompatible at the same concentrations, its cell viability was less than PMHA. This is due to the presence of the PEG coating in PMHA that provides higher cell compatibility.

The IC_50_ values for CUR@MHA, CUR@PMHA, and free curcumin in MCF-7 cell line were significantly lower than A549 cancer cells (*p* < 0.05), indicating higher cell toxicity against MCF-7 cells. Among the three cell lines, the highest cell viability after treatment with all tested materials was seen in normal MRC-5 cells. The IC_50_ value only was determined for CUR@PMHA (397 ± 2.5 *µ*g/mL), and it was not feasible to calculate for CUR@MHA, and free curcumin because of the treated cell viabilities were more than 50%. The IC_50_ of CUR@PMHA in the normal cells was significantly higher than A549 and MCF-7 cancer cells (*p* < 0.05), showing the lowest toxic effect of the CUR@PMHA on the normal MRC-5 cells and possible anticancer selectivity and safety. The IC_50_ results of other studies also showed higher cell toxicity of their curcumin-loaded nanomaterials and free curcumin on the cancer cells than the normal one [37, 44].

The IC_50_ values for both CUR@MHA and CUR@PMHA were significantly higher than free curcumin in A549 and MCF-7 cell lines (*p* < 0.05), indicating their higher cytotoxicity than free curcumin. This is due to the hydrophobicity of free curcumin that prevents the effective release of the drug in the medium. The finding is in agreement with the results of the studies that compared the cytotoxicity of other curcumin-loaded nanomaterials with free curcumin [37, 45, 46].

Between the CUR@MHA and CUR@PMHA, the IC_50_ was much lower for CUR@PMHA in both A549 and MCF-7 cells (*p* < 0.05). Therefore, the cell toxicity was higher for CUR@PMHA due to the PEG coating presence, which increases the curcumin hydrophilicity, allowing its higher release in the cell culture. The results of this study confirm the high performance of CUR@PMHA as a curcumin carrier nanosystem.

### 4.4. MRI

The signal dropping with increasing the iron concentration, linear relationship between 1/T2 amounts and Fe concentration, and high r2 relaxivity of PMHA (Figure 7) indicates the nanocomposite potential to act as an MRI negative contrast agent. The relaxivity of the nanocomposite (120 mM^−1^S^−1^) is similar to the r2 relaxivity of Endorem as a commercial MRI contrast agent. This finding confirms the high potential of PMHA to enhance the contrast of T2-weighted MR images.

The r2 relaxivity of PMHA nanocomposite in this study is higher than other HA, or PEG-coated nanostructures, including the mesoporous superparamagnetic hybrid silica/hydroxyapatite (MSFeHA) nanocomposite [32], iron oxide-hydroxyapatite (IO-HA) nanocomposite [33], Fe_3_O_4_@OA@PEG nanomicelles [47], and PEG surface-modified ultrasmall superparamagnetic iron oxide nanoparticles [48]. Different relaxivity values in these studies are related to the various sizes of the nanostructures, molecular weight of the PEG coating, and presence of other coating materials along with the HA or PEG.

Since PMHA nanocomposite consists of two coating materials around the magnetite nanoparticles, its r2 relaxivity is lower than the calcium phosphate coated iron oxide nanoparticles in another study [34]. The presence of HA and PEG coatings in PMHA leads to the increasing distance between the water protons and the magnetite nanoparticles. Therefore, the water protons experience a lower local magnetic field inhomogeneity in the presence of PMHA compared to the iron oxide nanoparticles with only a calcium phosphate coat, leading to a lower spin–spin interaction, T2 shortening, and relaxivity for PMHA. However, as noted, PMHA nanocomposite showed an r2 relaxivity comparable to the commercial iron oxide-based contrast agent. Additional PEG coating in PMHA provides a higher cytocompatibility for the nanocomposite than the calcium phosphate-coated iron oxide nanoparticles. Moreover, using an additional PEG coating around HA in PMHA can allow binding of functional groups and a safe intravenous administration of the nanocomposite in future in vivo studies.

## 5. Conclusion

In this study, the PEGylated magnetite/hydroxyapatite (PMHA) nanocomposite was successfully synthesized, characterized, and its r2 relaxivity, curcumin loading/release properties, and cell toxicity were investigated. The nanocomposite showed spherical shape, ultrasmall size, superparamagnetic behavior, high cytocompatibility, and high r2 relaxivity, implying its high potential to use as a T2-weighted contrast agent for MRI. Moreover, a high curcumin loading capacity and pH-dependent release profile with a considerable rate at the pH of tumor environment suggest that PMHA is also a promising candidate for delivery of curcumin as an anticancer drug. The results also confirmed the role of PEG coating in an efficient release of curcumin from CUR@PMHA. Based on the IC_50_ determination results, the significant toxic effect of CUR-PMHA on A549 and MCF-7 cancer cells was observed that be much higher in MCF-7 cells. The MTT results also indicated the significant cytotoxicity of CUR-PMHA compared to free curcumin.

Investigation of the PMHA nanocomposite potential for delivery of other hydrophobic chemotherapeutic drugs, and testing the results of this study for in vivo situations can be carried out in future studies.

## Data Availability

All of the data used to support the findings of this study are included within the article.
